# The Dynamics of Phosphorus Uptake and Remobilization during the Grain Development Period in Durum Wheat Plants

**DOI:** 10.3390/plants11081006

**Published:** 2022-04-07

**Authors:** Mohamed El Mazlouzi, Christian Morel, Thierry Robert, Coralie Chesseron, Christophe Salon, Jean-Yves Cornu, Alain Mollier

**Affiliations:** 1 INRAE, ISPA, Bordeaux Sciences Agro, 33140 Villenave d’Ornon, France; christian.morel@inrae.fr (C.M.); thierry.robert@inrae.fr (T.R.); coralie.chesseron@inrae.fr (C.C.); jean-yves.cornu@inrae.fr (J.-Y.C.); alain.mollier@inrae.fr (A.M.); 2 Univ. Bordeaux, UMR 1391 ISPA, 33000 Bordeaux, France; 3 INRAE, Agroécologie, AgroSup Dijon, University Bourgogne, University Bourgogne Franche-Comté, 21000 Dijon, France; christophe.salon@inrae.fr

**Keywords:** phosphorus, wheat grain, ^32^P tracer, grain P concentrations, P accumulation, post-anthesis P uptake, P partitioning

## Abstract

Post-anthesis phosphorus (P) uptake and the remobilization of the previously acquired P are the principal sources of grain P nutrition in wheat. However, how the acquired P reaches the grains and its partitioning at the whole plant level remain poorly understood. Here, the temporal dynamics of the newly acquired P in durum wheat organs and its allocation to grain were examined using pulse-chase ${}^{32}$P-labeling experiments at 5 and 14 days after anthesis. Durum wheat plants were grown hydroponically under high and low P supplies. Each labeling experiment lasted for 24 h. Plants were harvested 24, 48, and 96 h after labeling. Low and high P treatments significantly affected the allocation of the newly acquired P at the whole plant level. Three days (96 h) after the first ${}^{32}$P-labeling, 8% and 4% of the newly acquired P from exogenous solution were allocated to grains, 73% and 55% to the remainder aboveground organs, and 19% and 41% to the roots at low and high P supplies, respectively. Three days after the second labeling, the corresponding values were 48% and 20% in grains, 44% and 53% in the remainder aboveground organs, and 8% and 27% in roots at low and high P supplies, respectively. These results reveal that the dynamics of P allocation to grain was faster in plants grown under low P supply than under high supply. However, the obtained results also indicate that the origin of P accumulated in durum wheat grains was mainly from P remobilization with little contribution from post-anthesis P uptake. The present study emphasizes the role of vegetative organs as temporary storage of P taken up during the grain filling period before its final allocation to grains.

## 1. Introduction

Phosphorus (P) is an essential nutrient for all living organisms. In plants, it is required for energy transfer (ATP) and signal transduction as well as for the structure of nucleic acids and phospholipids [1]. Plants absorb P through the root cell transporters as orthophosphate ions (Pi) present in the soil solution [2,3]. In cereal crops, most of the absorbed P is allocated to the grains at maturity [4,5]. Batten [4] reported a P harvest index (the content of P in grain divided by total aboveground P content) of 30% to 90% in field-grown wheat. Exported P through harvest is regularly reintroduced in the soil stock by P fertilizers to sustain high crop productivity [6,7]. However, only 15–30% of applied fertilizer is taken up by crop roots during the first year after its application [8]. This low efficiency is due to the high fixation capacity of many soils, the low diffusion of Pi in the soil solution, and to the loss of Pi to the environment through leaching and soil erosion [3,8]. Moreover, the applied mineral P fertilizers are extracted from phosphate rock, which is a finite resource [8]. The demand for P fertilizer is expected to increase in the upcoming year as a result of the increasing population [8]. One possible strategy to reduce the amount of mineral P fertilizer is to reduce the export of P from the field by moderately reducing the concentration of P in the grains [7,9]. According to Lott et al. [10] estimates, the annual quantities of P exported with the harvested product (dry seed and grain) are equivalent to 85% of applied P fertilizers on a global scale. Therefore, moderately reducing P export from the field and improving P use efficiency in cropping systems require a better understanding of the mechanisms implicated in P accumulation in grains of cereal crops [9,11]. These mechanisms encompass post-anthesis P uptake, remobilization, and the pathways of its allocation to grains [11].

Plants have developed several mechanisms to acquire Pi and increase its use efficiency [12,13]. These mechanisms includes modification of root architecture and physiology (e.g., root exudation, secretion of phosphatases) to increase root exploration area and to mobilize more P from the soil [3]. Moreover, the availability of P is influenced by soil management practices and soil properties, such as pH, moisture, and microbial activity [13]. The primary goal of improving P uptake efficiency is to increase the ability of plants to recover P from soil and fertilizer [9,13]. The processes that control the availability of P and its uptake by plant roots have been well characterized during the early stages of plant development because the impact of P deficiency has a more notable negative consequence on crop productivity than P deficiency during later stages [14,15]. Nevertheless, a better understanding of the processes involved in the allocation of P to grains is necessary to reduce the accumulation of P in the grains during the reproductive phase [9]. In cereal crops, anthesis indicates the end of the vegetative stage and the beginning of the reproductive stage [16]. After anthesis, the developing grains are the terminal sink for carbon (C), proteins and nutrients [16,17]. During its growth period, the developing grain goes through three main phases: cell division followed by an expansion phase, a grain filling phase, and finally, a maturation phase [16]. The first two phases are the most important for grain weight and final grain yield [18]. Carbon supply to grains in wheat is mainly derived from assimilates during this period and from stem reserve that could provide up to 70% of total carbon [19]. Grain P demand is fulfilled by two sources: the internal remobilization of P from different plant organs and the concurrent P uptake from exogenous sources [12]. However, the contribution of these two sources to final grain P varies depending on genotypes, pre- and post-anthesis growing conditions, especially P and water availability [20,21]. The remobilization of P is reported to increase in response to P deficiency or during the grain filling period as a result of plant tissue senescence [22,23]. Given its mobility in phloem, P in older tissues can be easily remobilized and distributed within plant parts under P limited conditions and during senescence [1].

Grain reserves are the primary source of minerals and energy to seedling during the heterotrophic stage before root emergence [24,25,26]. Despite its great importance for germination and early plant development, grain P with concentrations ranging from 3 to 5 mg g${}^{-1}$ are often considered over the need for seedling development for many cereal crops [26,27,28]. To engineer crops with reduced grain P concentrations, it is possible to exploit the genotypic variations in grain P concentrations observed in many cereal crops, such as wheat [29], rice [30], and maize [31], and to manipulate the processes involved in the transport and the allocation of P to grains [11,32]. While the first strategy is long, and traditional breeding selection might lead to undesirable traits (e.g., low seedling vigor) and would be environmentally-dependent [26,30,33], the second strategy offers a better perspective and might be used for many crop species [11]. For example, the use of molecular and ${}^{32}$P-labeling approaches has advanced the knowledge regarding the pathways of P transport and accumulation in rice grains [33,34]. These studies have also led to the identification of low grain P rice by targeting a P transporter involved in the distribution of P to the developing grain [34]. Despite these major advances in understanding the P dynamics in rice, many uncertainties persist on how the newly absorbed P is transported to the grain in other cereal crops [34,35].Particularly, the regulation and the partitioning of post-anthesis P uptake and its accumulation in wheat grains remain unclear [11].

Tracer experiments using stable or radioactive isotopes are powerful tools to investigate nutrient fluxes at the whole plant level [36,37]. They have been used extensively to study nutrient dynamics during grain filling, especially for elements with several stable isotopes such as nitrogen and sulfur [38,39]. Previous quantitative studies of P dynamics in plants using P-tracer have generally been carried out at early growth stages [24,25]. For example, thanks to the labeling of exogenous nutrient solutions with ${}^{32}$P, Nadeem et al. [25] reported that maize roots started to take up exogenous P five days after sowing and they concluded that seed P reserves is required to supply maize seedling before root emergence. In contrast, little information is available on the dynamics of P during grain development in wheat.

The growing grains are the dominant sink for C and nutrients, including P during the post-anthesis period [22,40]. Before reaching the grains, the P goes through several steps, including exogenous Pi uptake, transport, and remobilization before the eventual delivery to the grains [32]. However, the dynamics of these events and the importance of post-anthesis P uptake in supplying the grain with P is unclear [11,32,41]. A better understanding of the mechanism involved in P accumulation in grains after anthesis could help identify key processes and potential means to improve crop P nutrition [11,32]. Yet, except in rice, only a few studies have investigated the dynamics of P uptake and partitioning during the grain development period in wheat.

The aim of the present study was to determine the short-term dynamics of P uptake and transport to the grains during the post-anthesis period in durum wheat. We hypothesized that during early grain development (e.g., cell division phase), little exogenous P would be allocated to the grain as a result of the low sink strength. In contrast, during the grain filling period, the remobilization of P from vegetative organs would contribute more to the allocated P to the grains as a result of the progress of plant senescence. In addition, it could be assumed that the intensity of P allocation to grains would be dependent on the plant P status.

## 2. Results

### 2.1. Plant Growth

**Figure 1:**
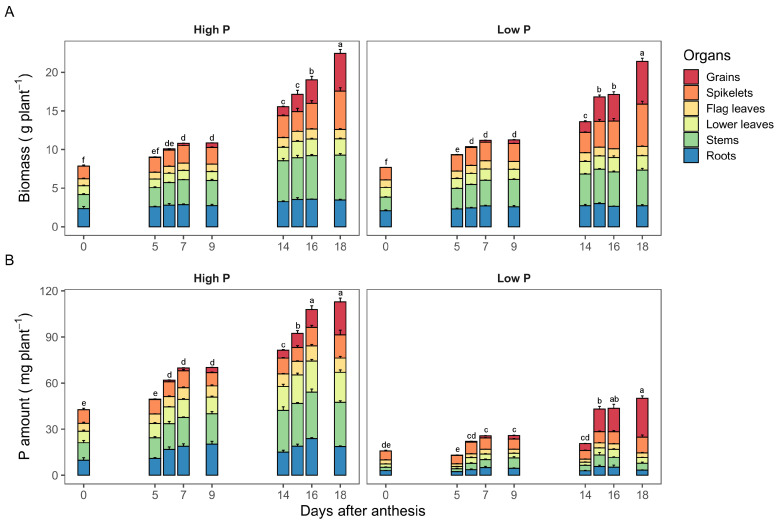
Biomass (**A**) and P amount (**B**) in different organs of durum wheat grown under high and low P supplies. Values are mean ± standard errors of three replicates. Different letters indicate significant differences between days for total biomass or P amount according to the LSD test at *p* < 0.05.

Durum wheat plants (cv. *Sculptur*) were grown with two levels of P supply to obtain plants with different P status. The plants were harvested at anthesis to determine the P status at this stage. Thereafter, two groups of plants were selected for the two labeling experiments as detailed in the Materials and Methods section (See Section 4). Total plant biomass did not differ significantly between high and low P supplies at anthesis. Thereafter, total biomass increased significantly all along the post-anthesis period. Between the anthesis and 18 days after anthesis (DAA), total plant biomass increased from 7.9 to 22.5 g plant${}^{-1}$ for high P plants and from 7.7 to 21.4 g plant${}^{-1}$ for low P plants (Figure 1A). A remarkable increase in total biomass was observed in high P plants starting from 14 DAA. A significant difference in total biomass between low P and high P plants was only observed at 14 DAA.

Biomass accumulation in grains did not vary significantly between high and low P supplies (Figure 1A). It started to increase from 5 DAA and continued during the grain filling period. Between 5 and 18 DAA, grain biomass increased from 0.05 to 4.9 g in high P plants and from 0.04 to 5.5 g in low P plants. The partitioning of biomass among plant organs was slightly different between high and low P supplies. Plants grown in low P supply allocated more biomass to grains (26%) than those grown under high P supply (22%). The proportion of total biomass allocated to roots and stems were lower in low P plants compared to high P plants, particularly during the grain filling stage.

### 2.2. Dynamics of P Accumulation among Durum Wheat Organs

Unlike biomass, durum wheat plants showed a contrasting pattern of P accumulation in response to P supply (Figure 1B). Plants grown at high P supply accumulated significantly higher P amounts than those grown at low P supply across harvest days. The total amount of P increased from 42.7 at anthesis to 112 mg P plant${}^{-1}$ at 18 DAA in the high P treatment, whereas it increased from 15.8 at anthesis to 50.2 mg P plant${}^{-1}$ at 18 DAA in the low P treatment. The increase in the total P amount in high P plants was mainly due to the increase of P amount in all organs. However, grains and spikelets were the main organs responsible for the increase of total P in low P plants.

The partitioning of P among plant organs was strongly affected by P treatments. For example, the proportion of P in spikelets at anthesis was 37% and 21% under low and high P supplies, respectively. At 18 DAA, this proportions were 50% in grain and 20% in spikelets in the low P treatment and only 19% in grain and 13% in spikelets in the high P treatment. However, the P amount in grain was not significantly different between treatments, i.e., 21.4 and 25.3 mg P at 18 DAA for high and low P, respectively.

**Figure 2:**
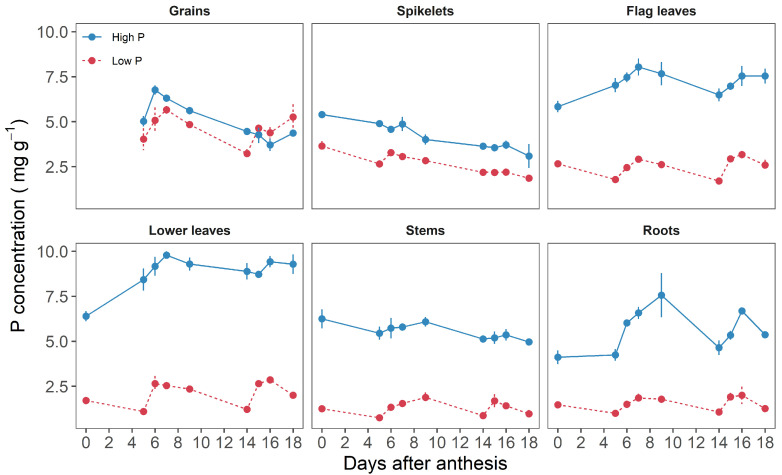
Phosphorus concentrations in durum wheat organs (Grains, spikelets, flag leaves, lower leaves, stems, and roots) during grain development (between anthesis and 18 days after anthesis). Plants were grown in a hydroponic culture with high or low P supply. Symbols are means of three replicates. Bars are standard errors.

In all plant organs, except grains, and regardless of the time elapsed after anthesis, high P plants had significantly greater P concentrations than low P plants, indicating that plants had different P statuses (Figure 2). Grain P concentrations had similar trends for both treatments and was only significantly different at 9 and 14 DAA. For high P plants, it increased from 5 at 5 DAA to 6.7 mg g${}^{-1}$ at 6 DAA and then decreased to 4.3 mg g${}^{-1}$ at 18 DAA. For low P plants, it started from 4 at 5 DAA to reach 5.2 mg g${}^{-1}$ at 18 DAA. The concentration of P in spikelets ranged from 3.6 to 1.9 and from 5.4 to 3 mg g${}^{-1}$ in low and high P supplies, respectively. Plants grown under low P supply had largely lower concentrations than those grown under high P supply (e.g., flag leaves 2.6 vs. 5.8 mg g${}^{-1}$ at anthesis, respectively). The highest P concentration was found in lower leaves of high P plants and in spikelets and grains for low P plants. The P concentrations in other organs were two to three times greater in high P plants in comparison to low P plants. The concentrations of P in leaves and roots were stable whereas they decreased slightly in spikelets and stems across P treatments.

### 2.3. The Dynamics of the Newly Acquired P (P${}_{exo}$) during the Post-Anthesis Period

**Figure 3:**
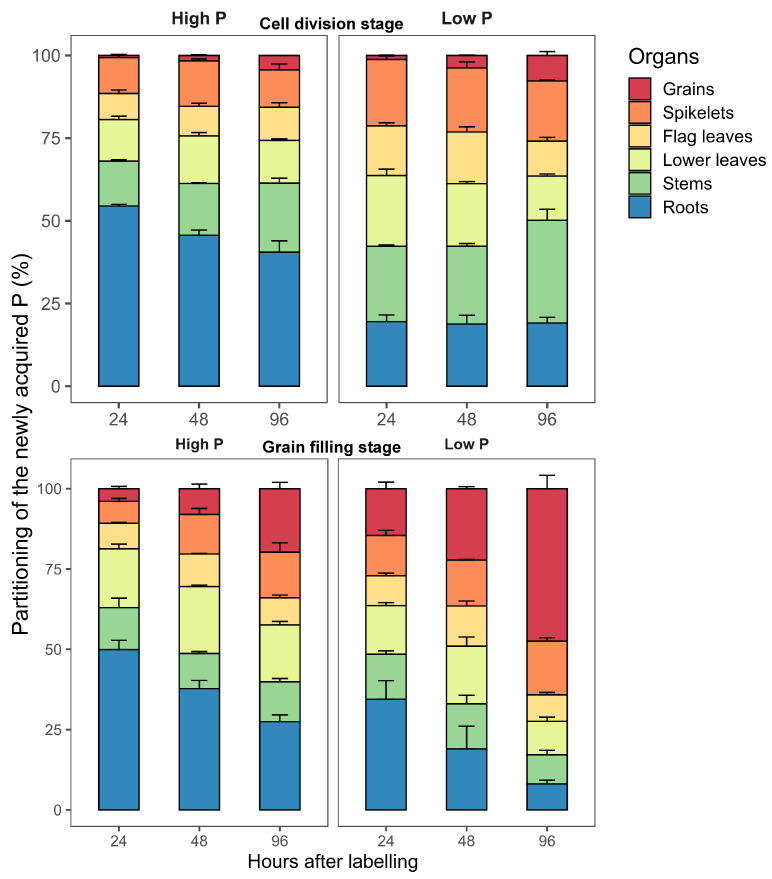
The partitioning of the newly acquired P in different durum wheat organs (grains, spikelets, flag leaves, lower leaves, stems, and roots) following ${}^{32}$P labeling at the cell division stage and grain filling stage. Values are the mean ± standard error of three replicates. Plants were grown in a hydroponic culture with high or low P supply. Each labeling experiment lasted 24 h.

Introducing orthophosphate ions, tagged with ${}^{32}$P, in the nutrient solutions allowed the determination of the dynamics of the P${}_{exo}$ and its partitioning and allocation among plant organs during grain development (Figure 3). The first experiment of ${}^{32}$P-labeling started at 5 DAA (cell division stage) and the second at 14 DAA (grain filling stage). Each labeling experiment lasted for 24 h. The amount of P${}_{exo}$ uptake after the 24 h of labeling was higher in the plants grown under high P supply in comparison to those grown under low P supply in both labeling (Table 1). While low P plants acquired the totality of P present in the nutrient solution (on average, 3.2 mg in the first ${}^{32}$P-labeling and 4.6 mg in the second ${}^{32}$P-labeling), there was greater variability among individual plants in the high P supply treatment (Table 1). For example, the total amount of P${}_{exo}$ in plants in the high P treatment was lower at 96 h after the first labeling compared to the total amount of P${}_{exo}$ in plants sampled at 24 h and 48 h after labeling. This might be related to the loss of P by efflux from the roots. Subsequently, the results are expressed as a percentage (%) relative to total exogenous P uptake (Figure 3).

#### 2.3.1. Partitioning of P${}_{exo}$ during the Cell Division Stage

After the first ${}^{32}$P-labeling experiment, the mean of P${}_{exo}$ for the three subsequent harvests, i.e., 24 h, 48 h, and 96, was 9.1 and 3.3 mg P plant${}^{-1}$ for high and low P, respectively (Table 1). The partitioning of P${}_{exo}$ among plant organs varied between P supply and across days after anthesis (Figure 3). In high P plants, 24 h after the first labeling (6 DAA), about 47% of the P${}_{exo}$ was recovered in the aboveground organs mainly in leaves (20%), stems (14%), and spikelets (11%). By 96 h after labeling, 60% of the P${}_{exo}$ was found in aboveground organs where stems (21%) and leaves (23%) were the primary sinks for P. The grain accumulated only 5% of the P${}_{exo}$. In contrast, following 24 h after labeling, the majority of the P${}_{exo}$ in low P plants (80%) was transported to the aboveground organs where 36% were found in leaves, 23% in stems and 20% in spikelets, whereas only 20% remained in the roots. After 96 h, the P${}_{exo}$ increased in stems (31%) and in grains (8%) indicating that in-between organs, P transfer had occurred.

#### 2.3.2. Partitioning of P${}_{exo}$ P during the Grain Filling Stage

During the grain filling stage, durum wheat plants continued to take up exogenous P from the nutrient solution in both P supplies. The mean of P${}_{exo}$ after 24 h of ${}^{32}$P-labeling was 8.9 and 4.6 mg P plant${}^{-1}$ for high and low P supplies, respectively (Table 1). In high P plants, half of P${}_{exo}$ was allocated to the aboveground organs whereas the other half was retained in roots immediately after the 24 h of ${}^{32}$P-labeling (Figure 3). The proportion of the P${}_{exo}$ in roots decreased progressively between 48 and 96 h after labeling with a concurrent increase in aboveground organs, indicating root to aboveground organ P transfer. The main sink organs for the P${}_{exo}$ was stems, lower leaves, spikelets and grains. In low P plants, 66% of the P${}_{exo}$ after 24 h of labeling was allocated to the aboveground organs. This proportion increased to 92% by 96 h after labeling. From 24 h to 96 h after labeling, the proportion of P${}_{exo}$ in vegetative organs declined while it continued to increase in grains and spikelets. It accounted for 48% in grains, followed by leaves (19%) and spikelets (14%) at 96 h after labeling. The relative reduction in the partitioning of the P${}_{exo}$ in roots and vegetative organs indicate that these organs serve as a transit for the post-anthesis P uptake before its transport to grains that represent the final sink of P in the plant.

### 2.4. Phosphorus Fluxes into the Developing Grains

**Figure 4:**
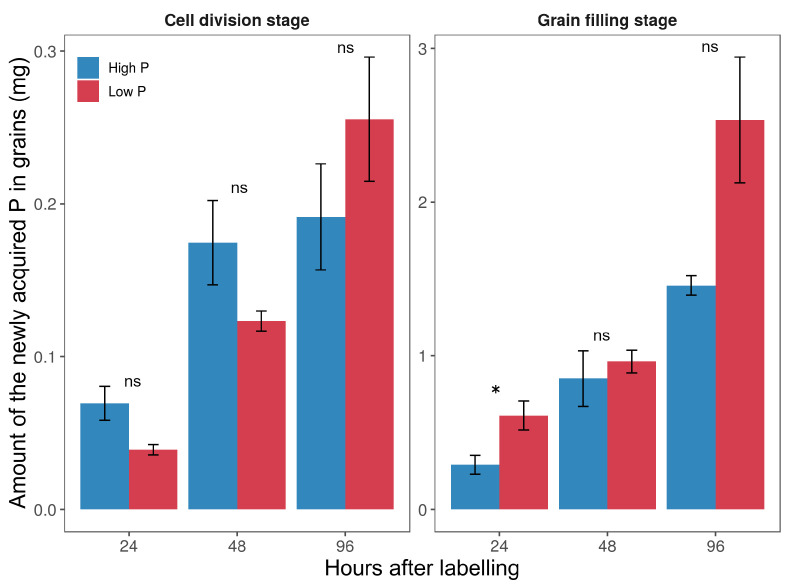
The amount of newly acquired P allocated to grains after the two labeling experiments in high and low P durum wheat plants; 24 h, 48 h, and 96 h represent hours after ${}^{32}$P labeling. Values are the mean ± standard error of three replicates. The asterisk indicates statistical significance between P supply (*p* < 0.05). ns: not significant. Note that the y-scale in the figure is different between the two stages for better visualization.

Despite the lower total P amount accumulating in the low P plants, the P amount in grains was not significantly different between the two P treatments in all harvest dates. The amount of P${}_{exo}$ allocated to grains increased progressively during grain development in both ${}^{32}$P-labeling experiments (Figure 4). It increased from 0.07 after 1 d in the first one to 0.3 in the second for high P supply, and from 0.04 to 0.6 mg P for low P supply. The only significant difference between the two levels of P supply in the allocation of the P${}_{exo}$ to grain was observed 24 h after the second labeling.

Although the newly acquired P uptake for high P plants was consistently greater than for low P plants, its allocation to grains was significantly lower, especially in the second labeling, as it can be seen from the ${}^{32}$PHI values (expressed in % to total exogenous P, Table 2). Between the first and the second labeling, it increased from 0.62% to 3.6% and from 1.2% to 14.6% after 24 h of labeling for high and low P supplies, respectively. At 96 h after the second labeling, about 48% of the P${}_{exo}$ was found in grains in the low P plants while only 19% in high P plants. Despite the significant allocation of the P${}_{exo}$ to grains, more than 90% of total P in grains comes from remobilization since ’recently taken up P’ only represents a small proportion of the total P in grains (Table 2). However, it seems that post-anthesis P uptake contributes more to grain P in low P plants than in high P plants.

## 3. Discussion

Determining the origin and P fluxes to grains is essential to identify potential targets to engineer crop plants with higher P use efficiency and reduce the unnecessary P export from the field. The dynamics of post-anthesis P uptake and its allocation to the grains was quantified by 24 h ${}^{32}$P-labeling experiments at two key stages of grain development: the cell division and expansion stage and the grain filling stage. The results of the two ${}^{32}$P-labeling experiments show the importance of vegetative organs as temporary places of P storage before subsequent transfer to the grains. This transfer seems to be influenced by plant P status and sink strength. A higher proportion of newly acquired P was allocated to the grains in the plants grown under low P supply than in the plants grown under high P supply. Our findings provide new insights into the short-term dynamics of P uptake and remobilization during grain development and into the possible pathways involved in P transport and accumulation in durum wheat grains.

### 3.1. Effects of P Status on the Partitioning of the Newly Acquired P among Plant Organs during Grain Development

The exogenous P supply had no significant impact on biomass accumulation, although the general trend was a minor increase in biomass for all plant organs in the high P supply (Figure 1A). This was possibly due to the continuous removing of vegetative tillers for both P treatments, as it was reported in previous studies [41,42]. Therefore, the supply of P can be considered sufficient in low P treatment to ensure the needs of the plant since the plant’s requirements have been reduced by keeping only four tillers. In contrast, durum wheat plants had different P status because the accumulation of P was significantly higher in high P treatment than in low P treatment (Figure 1B).

Plant P status affected the allocation of the P${}_{exo}$ among different plant organs. Regardless of the ${}^{32}$P-labeling dates, a higher proportion of P${}_{exo}$ was exported to the aboveground organs in low P plants in comparison to high P plants (Figure 4). Indeed, during early grain development, such at the cell division stage, P is needed to maintain metabolic activity in photosynthetic organs (e.g., leaves, spikelets) and to support grain growth [43,44,45]. The competition between these two processes can lead to different P partitioning patterns in high and low P plants among aboveground organs. While high P plants had ample P, suggesting the absence or low competition between vegetative organs and developing grains, this hypothesis cannot be excluded for low P plants and may explain the rapid allocation of P${}_{exo}$ to grain in this treatment. Jeong et al. [46] showed that P demand created by growing rice (*Oryza sativa* L.) grains can impair photosynthesis under limited P supply during early stages of grain development. However, it should be noted that P demand of growing grains is low during the early period of grain development. Furthermore, it was shown that the most accumulated P in durum wheat grains is loaded, starting from 10 days after anthesis [16,20]. During the grain filling stage, the grain becomes the main sink for assimilates and nutrients due to the progression of plant senescence. [17,40]. Thus, biomass increase occurring during this stage can be attributed to grain filling.

The obtained results show that the proportion of the P${}_{exo}$ allocated to grains is influenced by the plant P status since low P plants allocated more P${}_{exo}$ to grains than high P plants following 96 h after labeling on both labeling dates. These results are consistent with the initial hypothesis, assuming that the intensity of the P allocation to grains would be dependent on the plant P status. The difference between the two treatments may result from the rapid allocation of P to the grains in low P plants and/or from the limited distribution of P${}_{exo}$ to grains in the high P plants. In addition, the remobilized P seems to be satisfactory to meet the demand of the growing grains in high P plants. Modification of P distribution is one of the common responses to P limitations in cereal crops [47,48]. These modifications generally lead to higher allocation of C and P to the root, resulting in a higher root to shoot ratio in low P plants [48,49]. Moreover, a lower proportion of newly acquired P remained in the root following 96 h after the second labeling in comparison to the first labeling, where a higher proportion of newly acquired P was retained in the roots, particularly in high P plants (40%). This indicates that roots can act as active sinks for P during grain filling. In fact, roots continue to take up nutrients after anthesis despite the progressive decrease of their activity due to plant senescence [40]. In the short-term (6 h) P uptake experiment using ${}^{32}$P-tracer, Snapp and Lynch [50] showed that bean (*Phaseolus vulgaris* L.) roots retained over 80% of the acquired P under low P conditions while only 20% were retained at high P conditions. They suggested that bean roots retain P when exogenous P levels are low to sustain nutrient and water uptake as long as possible [50].

### 3.2. The Fate of the Newly Acquired P and the Mechanisms Implicated in P Transport and Accumulation in the Grains

The results show that even in a system with continuous exogenous P supply, as with hydroponic culture, the majority of the P absorbed during the post-anthesis period is not directly delivered to the grains, but temporarily stored in the organs with high transpiration flux such as leaves and spikelets. The short-term ${}^{32}$P-labeling used in this study allowed to trace the fate of the newly acquired P from the nutrient solution during grain development among durum wheat organs. Immediately after the end of the first labeling period (5 DAA), the grains received a little proportion (<2%) of the newly acquired P, although newly acquired P was present in spikelets and flag leaves (Figure 4). However, this proportion was much higher in the 24 h following the second labeling, particularly for the low P plants (14.6%). This proportion increased over time for both P supply, but it was more pronounced in low P plants than in high P plants. In the second labeling, the proportion of the newly acquired P found in the grains could be attributed to the redistribution from other organs, which may have already started during the 24 h of labeling time. These findings suggest that P taken up by roots during grain development is not directly transported to grains, but transit by vegetative organs first before reaching the final sinks. It is generally admitted that the acquired P by roots moves rapidly in the form of inorganic P to aboveground parts via xylem whereas its translocation within the plants occurs essentially via the phloem [2]. Consistent with our finding, wheat grains are characterized by the xylem discontinuity at the base of the grain [51], which mean that xylem-to-phloem transfer of P is required before entering the grain [52]. Furthermore, this discontinuity in the xylem may limit the transport of P with the transpiration stream, which is the principal driver of P transport in the xylem [53,54]. In wheat, the vascular systems in stems (particularly the nodes) are the locations where P exchanges from xylem to phloem, and are the most intense, allowing P to be directed from the transpiration stream to low transpiration organs, such as apexes and developing seeds [55,56]. The results obtained in the current study reinforce previous findings showing that the accumulation of P and phloem-mobile nutrients in cereal grains occurs mainly via the indirect transfer of newly absorbed and stored P in vegetative organs [35,52].

The faster allocation of P to grains in low P plants may also be associated with an earlier sink formation (grains) in low P plants in comparison to high P plants. Nutrient stress including P is shown to accelerate senescence and the development of grains in wheat and, thus, the remobilization of nutrients [23,57,58]. This is in line with findings by Snapp and Lynch [50], who found that leaf remobilization occurred earlier in low P bean plants in comparison to high P plants. Generally monocarpic senescence starts after anthesis and progresses during grain development [17]. This is consistent with the dynamics of P observed in roots, stems, or lowers leaves, which turned from sink to source for the newly acquired P during the the grain-filling period. This is also in agreement with the observed accumulation of newly acquired P in flag leaves and spikelets, which are organs with late senescence [17]. When the senescence is advanced, the remobilization of P stored in the vegetative organs dominates P fluxes at the whole plant level [17,20,23].

In the current study, grain P was mainly provided through the remobilization of endogenous P sources since P absorbed during the post-anthesis period accounted for only a very small fraction of the P accumulated in the grains (Table 2). The contribution of P uptake to grain P seems to be lower during the late stage of grain filling than at the beginning. This lower contribution may be associated with the progression of plant senescence and the increased remobilization that could meet grain P demand. Post-anthesis root P uptake capacity in wheat reported in the literature is considerably different and varies between genotypes and environmental conditions [20,21,59]. For example, Rose et al. [59] showed that wheat plant ceases P uptake after anthesis while Manske et al. [21] found that post-anthesis P uptake represented 48% of total P uptake at maturity. In contrast, many findings showed that wheat plants are heavily dependent on P remobilization from vegetative organs to fulfill grain P requirements [20,23,58]. By removing the post-anthesis P supply to durum wheat plants, El Mazlouzi et al. [20] demonstrated that P remobilization from senescence tissue was sufficient to supply the grain with P without affecting grain yield. In a recent study with the same cultivar, the remobilization of P reserves from spikelets and leaves to grains was estimated to represent more than 78% of the grain P at maturity in low P plants [41]. Similarly, withdrawing P from the nutrient solution during flag-leaf expansion did not influence grain P and yield in one-tiller wheat [58]. At low P supply, or when the demand for P is high, the stored P can be easily remobilized and transported to sink organs [12,60]. The high efficiency of P remobilization in wheat plants was suggested to be an important player in P fluxes to grains [32,41]. Around 50 to 90% of grain P can be attributed to P remobilization of endogenous P sources in wheat [4,23]. This was consistent with the decrease of P retention in roots and vegetative organs in low P plants observed in the present study. In addition to its high mobility in both phloem and xylem, P can be easily remobilized either from vacuoles, which is the main storage compartment in plant cells or following the breakdown on macromolecules, such as nucleic acid-P and phospholipids [61,62]. The ribonucleases (RNases) and acid phosphatases are shown to have a key role in liberating P from macromolecules during leaf senescence [43,62].

### 3.3. Routes for Lowering Grain P Accumulation in Durum Wheat and Future Perspectives

Although the values of post-anthesis P uptake could be overestimated due the fresh nutrient solution and the immediate availability of nutrients for root uptake (Table 1), the ${}^{32}$P-tracer study gives a clear overview on how the absorbed P is partitioned at the whole-plant level during grain development. It also demonstrates that when P supply is not limited, P uptake can occur during grain filling in durum wheat. Moreover, the remobilization of endogenous P sources was the main contributor of P accumulated in durum wheat grains. Theoretically, a reduction in grain P seems to be achievable by altering the allocation of the newly ’taken up’ P to grains or by decreasing P remobilization and transport to grains. The modification of the remobilization is not likely because it provides the majority of the nutrients accumulated in the grains. Mobile elements, such as zinc (Zn) and iron (Fe), originate from the remobilization of endogenous sources [63,64]. Additionally, about 66 to 82% of nitrogen (N) originates from remobilization of pre-anthesis stored N in winter wheat [65]. Any change in P remobilization through delaying senescence or by depressing the expression of enzymes implicated in P-containing molecular breakdown could negatively affect the mineral composition and the quality of the grains [11,22]. In fact, the mechanisms involved in senescence are not specific to P, and their alterations may impact other elements [11]. For example, the reduction in the expression of the NAM-B1 transcription factor, which play a crucial role in the remobilization of nutrients to grains during leaf senescence, lead to a decrease in P concentration and also decrease the concentration of N, Zn, and Fe [66]. Additionally, the downregulation of a transcription factor that reduces leaf senescence (e.g., GPC) resulted in reductions of grain Zn and Fe content in tetraploid wheat [67]. Any change of the senescence-related mechanism should take into account grain N because the grain protein content is the primary quality indicator that determines the end-use of wheat. The study of the plant and grain ionome could provide interesting insight, given the complexity of interactions that exist among nutrients in plant [37,68].

Since the majority of P accumulated in grain comes from remobilization and that P taken up after flowering does not provide a major contribution to the accumulated P in the grain, then modification of transporters specifically involved in P redistribution, originating from newly absorbed or remobilized P, could be a potential target for reducing P accumulation in grain in wheat. Accordingly, Yamaji et al. [34] showed that the SULTR-like P distribution transporter (SPDT), which is located in the stem nodes, operates as a switch for P distribution to rice grains. By knocking out the *SPDT* gene, the authors demonstrated that P accumulation in grains can be lowered by up to 20% by reducing the distribution of P to developing rice grains [34]. More importantly, the reduction in grain P was accompanied by a 30% decrease in phytate concentration with no change in grain yield. The authors also reported an increase in the bioavailability of zinc and iron [34]. In fact, phytic acid is considered an anti-nutrient agent because of its capacity to bind micronutrients, such as zinc and iron, and make them non-available to monogastric animals [6]. In durum wheat, the molecular players involved in the regulation of P transport to the grain are not yet clearly identified. Characterization of specific P transport systems in the nodes is a promising approach to reduce the unnecessary P export and improve the nutritional quality of grains. Given their similarity in grain histology, durum and bread wheat share the same pathways leading to P accumulation in the grain. Any progress in reducing P accumulation in the grain of durum wheat (especially phytate content) would more likely be transferred to other wheat.

## 4. Materials and Methods

### 4.1. Plant Growth Conditions

Durum wheat plants were grown hydroponically as previously described in El Mazlouzi et al. [41]. Uniform seeds of *Triticum durum* cv. *Sculptur* (RAGT Semences, Rodez, France) were disinfected using 6% (*v*/*v*) sodium hypochlorite (H${}_{2}$O${}_{2}$) and germinated in moist paper for 3 days at 25 °C. Thereafter, germinated seeds were placed in 50 mL Falcon${}^{®}$ tubes filled with modified Hoagland nutrient solution (25% strength for macronutrients and full strength micronutrients). Two weeks after germination, 72 uniform wheat seedlings were then transferred to individual pots containing 5.5 L of modified Hoagland’s nutrient solution consisting of: 0.125 mM KH${}_{2}$PO${}_{4}$, 0.625 mM KNO${}_{3}$, 0.85 mM KCl, 1.25 mM Ca(NO${}_{3}$)${}_{2}$, 0.5 mM MgSO${}_{4}$, 46.25 µM H${}_{3}$BO${}_{3}$, 1 µM MnCl${}_{2}$, 10 µM ZnSO${}_{4}$, 2 µM CuSO${}_{4}$, 0.03 µM (NH${}_{4}$)${}_{6}$Mo${}_{7}$O${}_{24}$, 100 µM NaFe EDTA, 25 µM HEDTA, 2 mM MES buffer, and 50 mg L${}^{-1}$ SiO${}_{2}$. To obtain plants of different P nutritional statuses, two levels of P supply (as KH${}_{2}$PO${}_{4}$, Merck) were applied, starting from the three-leaf stage: 0.025 mM KH${}_{2}$PO${}_{4}$ (low P supply) and 0.125 mM KH${}_{2}$PO${}_{4}$ (high P supply). To compensate for the difference in the K supplied, KCl was added to the lowest P treatment. The initial pH was adjusted to 6 ± 0.5 with KOH. Aeration and renewal of the nutrient solution was maintained by the overflow-type system. The pots were arranged in a randomized block design (18 plants per block). To prevent the high tillering rate associated with hydroponic culture, only the first four tillers were allowed to grow.

Plants were grown under greenhouse conditions, with a photoperiod of 16 h/8 h day/night and an average relative air humidity of 64%. Air temperature and relative humidity were recorded every 30 min using a data logger (CR32X sensor, Campbell Scientific, Logan, UT, USA). The plants were illuminated with an average of 450 µmol photons m${}^{-2}$ s${}^{-1}$, provided by natural light supplemented with LED lamps (model LED, Ledlyt, Pessac, France).

### 4.2. Plant Sampling and Measurements

**Figure 5:**
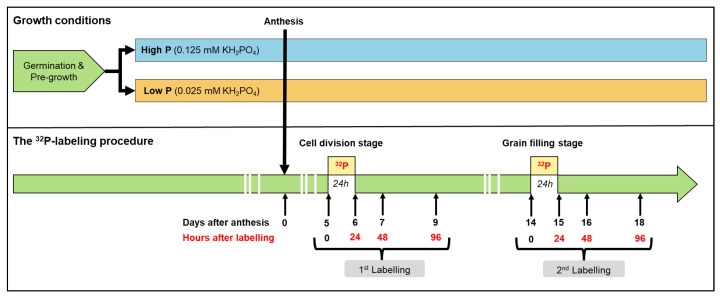
The experimental design used for plant growth and ${}^{32}$P labeling. Durum wheat plants were grown hydroponically under high and low P supplies under greenhouse conditions. The ${}^{32}$P labeling experiments were performed at 5 and 14 days after anthesis and lasted for 24 h. For labeling, plants were harvested after 24, 48, and 96 h after labeling. Three plant replicates were sampled at each harvest date.

The timeline of plant sampling and labeling is provided in Figure 5. After head emergence, the plants were individually monitored to determine the anthesis date. For both treatments, three plant replicates (n = 3) were harvested at anthesis and at 5, 6, 7, 9, 14, 15, 16, and 18 days after anthesis (DAA). At each harvest date, plants were separated into grains, spikelets (including rachis), flag leaves, lower leaves, stems (including peduncles), and roots. Root systems were rinsed with deionized water and blotted with paper towels to remove surface-bound nutrient solutions. For each plant, organs from the four tillers were pooled and then oven-dried at 60 °C for 72 h and their dry weights were determined.

### 4.3. Plant Labeling with ${}^{32}$P

The labeling experiments with ${}^{32}$P (half-life 14.3 days) were performed at two key stages of grain development: the first at 5 DAA (cell division phase) and the second at 14 DAA (grain filling) [16] (Figure 5). On the day of the first labeling, stock nutrient solution (54 L for each treatment) with the same composition as previously described was prepared and spiked at approximately 6 and 13 MBq of ${}^{32}$P radioactivity of orthophosphate ions (NEX054010MC, PerkinElmer SAS, France) for low and high P supplies, respectively. Thereafter, 18 plants (9 per treatment) were transferred to an adjacent greenhouse dedicated to handling radioisotopes using the same conditions specified above. Labeling experiments were initiated 8 h after the start of the photoperiod. The plants remained in the labeling solution for 24 h. Immediately after the end of the labeling period, 6 labeled plants (3 per treatment) were harvested and separated as detailed above. The remaining 12 plants were placed in a fresh unlabeled nutrient solution to continue their development under the same conditions until harvest. Before transferring plants to the unlabeled solution, roots were bathed and carefully rinsed three times with deionized water to eliminate the adhered ${}^{32}$P from root surface. The 12 remaining plants were harvested at 48 h and 96 h after labeling (i.e., 6 and 9 days after anthesis). The second ${}^{32}$P labeling at the grain filling stage, starting from 14 DAA, was carried out following the same procedure as the first one (Figure 5).

### 4.4. Phosphorus Concentration, ${}^{32}$P Analysis, and Partitioning Indices

Phosphorus concentration (mg g${}^{-1}$) in plant organs was determined using the malachite green colorimetric method at 610 nm [69]. Briefly, sub-samples of the plant organs were ashed in a muffle at 550 °C for 5 h. Ashes were digested in 2.5 mL of HNO${}_{3}$ and then washed with 5 mL of ultra-pure water. Digests were evaporated until only a few drops were left. Following evaporation, the samples were filtered and the volume was made up to 50 mL with ultra-pure water and used to determine the P concentration and ${}^{32}$P activity. For a given plant organ, the amount of P was calculated by multiplying the dry weight and its measured P concentration. The total amount per plant was calculated as the sum of the amount of P in all plant organs.

Radioactivity in plant samples and in the nutrient solution were measured using a liquid scintillation counter (Tri-Carb 2100TR, Packard BioScience). For each measurement, 1 mL of filtered samples was mixed with 3 mL of a scintillation cocktail (Insta-gel Plus Packard, Perkin-Elmer, Waltham, MA, USA) and measured for two minutes. All radioactivity measurements were corrected for the radioactive decay to a single time point. Assuming that no fractionation between ${}^{31}$P and ${}^{32}$P occurred during root P uptake and P transport within the plant, the exogenous P (P${}_{exo}$) in each plant organ derived from the exogenous nutrient solution during the labeling period was calculated according to the following formula, as described in Morel and Fardeau [70]:

$$\frac{R_{t}}{P_{ns}}=\frac{r_{t}}{P_{exo}}=>P_{{}_{exo}}=P_{ns}\times \frac{r_{t}}{R_{t}}$$

where R${}_{t}$/P${}_{ns}$ is the specific activity measured at time t in the nutrient solution, r${}_{t}$ is ${}^{32}$P activity measured in the plant samples at harvest time t after labeling. In the following sections, the exogenous P taken up during the 24 h of labeling (estimated from ${}^{32}$P measurements) will be referred to as the newly acquired P or P${}_{exo}$. The total amount of P${}_{exo}$ after the 24 h of ${}^{32}$P labeling was calculated as the sum of P${}_{exo}$ found in all plant organs.

### 4.5. Calculation of P Partitioning and Remobilization Indices

Two indicators were calculated to follow the partitioning and the allocation of the newly acquired P absorbed during the grain development period: PHI and ${}^{32}$PHI. The P harvest index (expressed in %, PHI) was calculated as the amount of P in the grains divided by the total amount of P in the other plant organs, including the roots. The ${}^{32}$PHI refers to the P${}_{exo}$ in the grain divided by the total P${}_{exo}$ in the whole plant. The percent contribution of P${}_{exo}$ to the accumulated P in the grain at each harvest date was calculated as the P${}_{exo}$ in the grains divided by the total amount of P in the grains. The amount of P remobilized from vegetative organs to grain P was calculated by the difference between grain P minus P${}_{exo}$ in the grain.

### 4.6. Statistical Analyses

All statistical analyses were conducted with R statistical software version 3.4.4 [71]. Data were reported as the mean ± standard error (SE) of the three replicates (n = 3). Significant differences between measured parameters (e.g., biomass, P amount) were tested across harvest days for each P treatment using a two-way ANOVA (*p* < 0.05). When ANOVA indicated a significant difference among harvest days or treatment, a multiple comparison test was carried out using the least significant difference test (*p* < 0.05). Prior to analyses of variance, normality and homogeneity of variance were checked.

## 5. Conclusions

The results of the present study revealed two main findings. Firstly, it showed that the transfer of P taken up during the post-anthesis period to grains was influenced by plant P status and sink strength, as low P plants allocated more newly acquired P to grains than high P plants. Secondly, the P taken up by roots during the post-anthesis period was not directly allocated to grains. It was temporarily stored in the vegetative organs (leaves, spikelets, and stems) before being reallocated to developing grains. This important result suggests that grain P filling is driven by indirect P fluxes from previously absorbed P stored in vegetative organs. Finally, since P absorbed after anthesis is not a major contributor to grain P accumulation, modification of transporters specifically involved in P redistribution, whether newly absorbed or remobilized P, may be a potential target for decreasing grain P accumulation in durum wheat. Therefore, the transport systems involved in P redistribution need to be studied to selectively modify its allocation to grains.

## Figures and Tables

**Table 1:** Total P amount (mg P plant${}^{-1}$) derived from exogenous P uptake during the two labeling experiments. Each labeling experiment lasted for 24 h. Values are mean ± standard errors of three replicates. HAL: hours after labeling. The asterisk represents a significant difference between high and low P treatments (*p* < 0.05). ns: not significant.

	Total Amount of Exogenous P (mg plant${}^{-1}$)		
Labeling	HAL	High P	Low P
First ${}^{32}$P-labeling (grain division stage)	24 h	11.3 ± 2.5	3.2 ± 0.1 *
	48 h	11.3 ± 2.5	3.2 ± 0.1 *
	96 h	4.4 ± 0.8	3.3 ± 0.1 ${}^{\mathrm{ns}}$
Second ${}^{32}$P-labeling (grain filling stage)	24 h	8 ± 2.7	4.2 ± 0.1 ${}^{\mathrm{ns}}$
	48 h	11.2 ± 2.5	4.3 ± 0.2 ${}^{\mathrm{ns}}$
	96 h	7.6 ± 1.2	5.3 ± 0.4 ${}^{\mathrm{ns}}$

**Table 2:** Phosphorus harvest index (PHI), ${}^{32}$PHI, and the contributions of newly acquired P to the accumulated P in the grains of durum wheat grown under high and low P supplies. DAA: day after anthesis. DAL: day after labeling. The asterisks *, ** and *** indicate significant difference at *p* < 0.05, *p* < 0.01 and *p* < 0.001 between high and low P treatments, respectively. ns: not significant.

Labeling	P Indexes	DAA (HAL)	High P	Low P
First ${}^{32}$P-labeling (grain division stage)	PHI (%)	6 (24 h)	1.7	2.4 ${}^{\mathrm{ns}}$
		7 (48 h)	2.6	5.3 **
		9 (96 h)	4.9	8.7 *
	${}^{32}$PHI (%)	6 (24 h)	0.6	1.2 *
		7 (48 h)	1.5	3.7 **
		9 (96 h)	4.3	7.9 *
	Contribution of the P${}_{exo}$ to total grain P (%)	6 (24 h)	6.6	7.5 ${}^{\mathrm{ns}}$
		7 (48 h)	9.7	9.1 ${}^{\mathrm{ns}}$
		9 (96 h)	5.6	11.4 **
Second ${}^{32}$P-labeling (grain filling stage)	PHI (%)	15 (24 h)	10	34.3 **
		16 (48 h)	10.7	35.1 **
		18 (96 h)	19	50.5 **
	${}^{32}$PHI (%)	15 (24 h)	3.6	14.6 **
		16 (48 h)	7.6	22.2 ***
		18 (96 h)	19.2	48.0 **
	Contribution of the P${}_{exo}$ to total grain P (%)	15 (24 h)	3.2	4.1 ${}^{\mathrm{ns}}$
		16 (48 h)	7.4	6.3 ${}^{\mathrm{ns}}$
		18 (96 h)	6.8	10.0 ${}^{\mathrm{ns}}$

## Data Availability

The data presented in this study are available upon request from the corresponding author.
